# The Association between E-Cigarette Price and TV Advertising and the Sales of Smokeless Tobacco Products in the USA

**DOI:** 10.3390/ijerph18136795

**Published:** 2021-06-24

**Authors:** Yu Wang, Zongshuan Duan, Sherry L. Emery, Yoonsang Kim, Frank J. Chaloupka, Jidong Huang

**Affiliations:** 1 School of Public Health, Georgia State University, Atlanta, GA 30303, USA; ywang145@student.gsu.edu (Y.W.); zduan3@student.gsu.edu (Z.D.); 2 NORC at The University of Chicago, Chicago, IL 60637, USA; emery-sherry@norc.org (S.L.E.); kim-yoonsang@norc.org (Y.K.); 3 School of Public Health, University of Illinois at Chicago, Chicago, IL 60612, USA; fjc@uic.edu

**Keywords:** e-cigarettes, smokeless tobacco, chewing loose leaf, moist snuff, snus, cigarettes, price, TV advertising, price elasticity

## Abstract

This study aims to examine how e-cigarette prices and advertising, key determinants of e-cigarette demand, are associated with the demand for smokeless tobacco (SLT) products in the US. Market-level sales and price data by year (2010–2017), quarter, and type of retail store were compiled from Nielsen retail store scanner database. E-cigarette TV advertising ratings data were compiled from Kantar Media. Four-way (market, year, quarter, store type) fixed-effect models were used to estimate the associations between e-cigarette price and TV advertising and sales of SLT products (chewing loose leaf, moist snuff, and snus). Our results showed that a 1% rise in own price was associated with a reduction in sales by 1.8% for chewing loose leaf, 1.6% for moist snuff, and 2.2% for snus, respectively. In addition, a 1% rise in disposable e-cigarette price was associated with 0.3% and 0.6% increased sales for moist snuff and snus, respectively. The association between e-cigarette TV advertising and SLT product sales was not significant. Our results suggest that disposable e-cigarettes and certain SLT products (moist snuff and snus) are potential substitutes. Policies aiming to regulate e-cigarette use and sales need to consider their potential link with the demand for SLT products.

## 1. Introduction

Smokeless tobacco (SLT) products in the US market can be categorized into three broad types, chewing tobacco, snuff, and dissolvable SLT products [1]. Chewing tobacco includes loose leaf, plug, twist, and other chewing tobacco products, with loose leaf being the most popular subtype in the US market [1,2]. Snuff is finely ground tobacco, which can be either dry, moist, or packaged in pouches or packets. Moist snuff is the most consumed SLT product in the US, accounting for approximately 90% of all SLT product sales between 2011 and 2019 [2]. The US snus is a relatively new SLT product, which is packaged in small ready-to-use pouches filled with moist snuff that does not require spitting [1]. Dissolvable SLT products, including strips, tablets, and sticks, which can dissolve slowly in the mouth, account for a very small (less than 0.01%) market share in the US [1,2,3]. In addition to containing nicotine, which could lead to addiction, SLT products also contain other components that are detrimental to users’ health [4,5]. Current evidence suggests that SLT use is associated with elevated risks of oral, esophageal, and pancreatic cancer, gum disease and tooth loss, and cardiovascular disorders [4,5,6,7,8].

Findings from previous studies suggest that SLT products are designed to appeal to smokers as a more socially acceptable nicotine delivery alternative due to the concerns about the health risks that cigarettes impose on both smokers themselves and bystanders [9,10]. Content analyses of the marketing materials from print media and social media find that SLT products are frequently promoted as a way to maintain nicotine consumption in places/times in which cigarette smoking is prohibited, and as a less harmful alternative to cigarettes [11,12,13,14]. In October 2019, the US Food and Drug Administration (FDA) granted modified risk orders to eight snus SLT products (under the “General” brand) produced by Swedish Match USA, Inc. [15]. These modified risk orders gave permission to Swedish Match USA, Inc. to market these eight specific snus products in the US with the claim “Using General Snus instead of cigarettes puts you at a lower risk of mouth cancer, heart disease, lung cancer, stroke, emphysema, and chronic bronchitis” [15]. This was the first time in FDA’s history that the marketing of a tobacco product had been authorized through the modified risk tobacco product (MRTP) pathway [15].

Similar to SLT products, e-cigarettes, a new and emerging product category that encompasses a variety of electronic nicotine delivery products, are also promoted as a less harmful alternative to combustible cigarettes [16,17,18]. For example, previous studies documented the presence of claims that e-cigarettes are a healthier and safer alternative to cigarettes and a smoking cessation tool across a variety of marketing channels [16,17,19]. However, such claims have not been approved by the US FDA.

The use of e-cigarettes has increased substantially in the US in the past decade. For example, previous studies showed that monthly e-cigarette sales increased by almost 300% (from 5.6 million units to 22.0 million units) between November 2016 and August 2019 [20]. According to the most recent data and the best data available, the prevalence of past-30-day use of e-cigarettes increased from 1.5% in 2011 to 19.6% in 2020 among high school students [21,22], and from 0.8% in 2011 to 3.2% in 2018 among adults [23,24]. During the same period, the prevalence of past-30-day use of SLT products declined from 7.9% in 2011 to 4.8% in 2019 among high school students [22,25], and from 3.6% in 2012 to 2.4% in 2019 among adults [4,26]. In addition, the sales data showed that SLT product sales leveled off or decreased since e-cigarettes became popular in the US market around 2016 [2].

Given that both e-cigarettes and SLT products are promoted as less harmful alternatives to cigarettes, the substantial growth of e-cigarette use in recent years may have diverted the use of SLT products among some tobacco users. Despite significant policy implications, the evidence on the potential impact of e-cigarette sales on the demand for SLT products is surprisingly scarce. Evidence from empirical studies is needed to examine the relationship between e-cigarettes and SLT products. This study is designed to fill this critical research gap by investigating whether, and to what extent, the sales of e-cigarettes are associated with the sales of SLT products in the US. Since e-cigarette sales and SLT product sales within the same retail market are likely to be endogenous, we use two key factors influencing the demand for e-cigarettes, e-cigarette price and TV advertising, in our analyses to estimate the association between e-cigarette sales and SLT product sales. Previous studies demonstrated that the price of e-cigarettes plays an important role in determining e-cigarette sales [27,28]. Additionally, unlike advertising of SLT products, which had been banned from television and radio since the statutory broadcast ban became effective in 1986 [29], the advertising of e-cigarettes remained largely unregulated until very recently [30]. Previous studies found that the substantial growth of e-cigarette awareness and usage in recent years in the US was at least partially attributable to the successful advertising campaigns by the e-cigarette industry [16,17,31].

This study aims to examine the associations between e-cigarette price, TV advertising, and the sales of SLT products, by linking the market-level SLT product sales data with e-cigarette and SLT product price data, and e-cigarette TV advertising ratings data, taking advantage of the variations in e-cigarette prices and e-cigarette TV advertising over time within the same retail market. Our study is designed to estimate the own-price elasticity of SLT products, cross-price elasticity of SLT products with regard to e-cigarettes and cigarettes, and the indirect effect of e-cigarette TV advertising on the demand for SLT products via its direct impact on e-cigarette sales.

## 2. Materials and Methods

### 2.1. Data

Study data—quarterly prices and sales data for SLT products, e-cigarettes, and cigarettes from the first quarter (Q1) of 2010 to the last quarter (Q4) of 2017—were obtained from the Nielsen Retail Store (NRS) scanner database (The Nielsen Company, LLC, New York, NY, USA), a database that tracks product sales and prices occurring in Nielsen participating retailers, including convenience stores (CV), and food, drug, and mass stores (FDM). Sales and prices of SLT products, e-cigarettes, and cigarettes were recorded at the UPC (universal product code) level. The UPC is a unique 12-digit code representing a specific product (for example, the UPC for a single-device package of the NJOY King disposable e-cigarette (NJOY, LLC, Scottsdale, AZ, USA) with mint flavor is 001068701473). Each product brand can have hundreds of different UPCs that capture the products with different characteristics (for example, nicotine content, flavor, package size, etc.), which all fall within the same product brand. There are 52 Nielsen-defined retail markets in the NRS scanner database that cover 44 states and the District of Columbia in the US. A Nielsen-defined retail market refers to the totality of all Nielsen participating retailers located in a delineated area, consisting of a cluster of geographically contiguous counties surrounding a major city, after which it is usually named.

Quarterly contemporaneous e-cigarette TV advertising ratings data were retrieved from the Kantar Media Stradegy^TM^ (Kantar Group, London, UK) database, a database created and maintained by the Kantar Group, which tracks the advertising exposure and advertising expenditures for more than 3 million product brands. Kantar Media advertising data are collected from 210 designated market areas (DMA) in the US. Similar to a Nielsen-defined retail market, each DMA also refers to a media market consisting of a cluster of geographically contiguous counties. However, a media market is always smaller than a retail market, and always fully contained within a retail market. Details on how the 210 DMA are mapped to the 52 retail markets are described in the next section. We retrieved the e-cigarette TV advertising ratings data by searching the Kantar Media Stradegy^TM^ database using a list of e-cigarette keywords. Details on the e-cigarette keywords on this list and how e-cigarette TV advertising data were retrieved from the Kantar Media Stradegy^TM^ database were described in a previous study [32]. This study analyzed aggregated sales, price, and advertising data, and was determined by the IRB review board to be non-human subject research.

### 2.2. Measures

#### 2.2.1. Per Capita Sales of SLT Products

We examined, separately, three categories of SLT products (chewing loose leaf, moist snuff, and snus) that were most popular in the US market, given the considerable differences across these three types of products in terms of product characteristics and patterns of use. Quarterly sales volumes measured by ounces for each category of SLT products were first calculated by aggregating the sales units of this category of SLT products sold in a specific store type (CV/FDM), in a specific retail market, and in a specific year/quarter. Per capita sales of this category of SLT products in a given market/store type/year/quarter were then calculated by dividing the aggregated total sales volume of this category of SLT products in this retail market/store type by the estimated total population resided in this retail market in the same year/quarter. The total population for each market for a given year/quarter was generated by summing up the county population of all counties located within this retail market. The quarterly county population was extrapolated using the yearly county population data, measured on 1 July (the mid-point) each year, which were retrieved from the US Census Bureau, assuming linear population growth over time [33].

#### 2.2.2. Inflation-Adjusted SLT, E-Cigarette, and Cigarette Prices

The average product price for a given market/store type/year/quarter was constructed by dividing the total sales dollars of one category of SLT products in a given market/store type/year/quarter by the total sales volume, measured by ounces, for this category of products for the same market/store type/year/quarter. The average price was then inflation-adjusted using the consumer price index (CPI) obtained from the Bureau of Labor Statistics [34]. The base period for the inflation adjustment was the fourth quarter of 2017. For example, the average price of snus sold in convenience stores in Atlanta market in 2016 Q3 was constructed by dividing the total sales dollars of snus sold in convenience stores in Atlanta market in 2016 Q3 ($5,072,142) by the total sales volume of snus sold in convenience stores in Atlanta market in 2016 Q3 (1,297,908 ounces). The price was calculated as $3.91 per ounce in 2016 Q3 dollars, with inflation adjustment, it was equivalent to $4.00 per ounce in 2017 Q4 dollars. Prices for disposable e-cigarettes and cigarettes were constructed using the same method, except that the sales volumes for e-cigarettes and cigarettes were measured by pieces and sticks, respectively. We did not include the prices of reusable e-cigarettes in our models since they included a wide variety of devices that differ drastically in shape, form, function, utility, and cost, making the average price per piece an inconsistent measure across different types of reusable e-cigarettes. In addition, due to the rapid evolution of reusable e-cigarette devices from 2010 to 2017, the average price for one reusable e-cigarette device in recent years may not be comparable to that of an earlier generation of reusable e-cigarette devices.

#### 2.2.3. E-Cigarette TV Ratings

The TV ratings (gross rating points (GRPs)) were used to measure the e-cigarette TV advertising exposure among US households. Quarterly GRPs were the multiplication of the percentage of households that watched an advertisement and the average number of times this advertisement was watched by these households within one quarter [35]. For example, if an advertisement were watched by 25% of households in a DMA for an average of 4 times in a quarter, the quarterly GRPs for this advertisement would be 100 in this DMA. Quarterly e-cigarette TV ratings for each DMA were calculated by summing up the household GRPs of all e-cigarette products’ advertising in that year, quarter, and DMA.

TV ratings data from the 210 DMA were mapped to the 52 retail markets using the state and county identifiers. All counties within the same DMA were assumed to have the same TV ratings for a given year/quarter. Each county’s TV ratings were matched to a retail market using the state and county identifiers. County population weighted GRPs were then calculated for each retail market. For any retail market, if its quarterly TV ratings value was less than 1, it was recoded to 1, the reason for this being that if the quarterly GRPs were less than 1, it means that less than one percent of the households in this market were exposed to e-cigarette advertising once during the entire quarter. This level of exposure is extremely small and close to no exposure at all. Since the distribution of TV ratings in our analysis is skewed, log transformation is needed for regression analysis. This recoding technique not only avoids the large negative values due to the log form of TV ratings (particularly when the ratings are less than 1 and close to 0), which could distort the regression results, but also takes into account the actual meaning of the TV ratings, with the value of 0 (after log transformation) referring to no or minimum level of advertising exposure.

### 2.3. Statistical Analyses

Data management and analyses were conducted using Stata/SE 15 (StataCorp LLC, College Station, TX, USA). The trends of average prices and sales volumes of chewing loose leaf, moist snuff, and snus in the US from 2010 Q1 to 2017 Q4 were presented. Models that included the year, quarter, market, and store type fixed effects were used to estimate: (1) the own-price elasticities for the three categories of SLT products; (2) the cross-price elasticities of SLT products with regard to disposable e-cigarettes and cigarettes, and the cross-price elasticities within the SLT product categories; and (3) the association between e-cigarette TV advertising and SLT product sales. Standard errors were clustered at the level of store type by market level to account for the potential correlations of sales within the same store type and market over time. In addition, Hausman tests were conducted to check if the error term was significantly associated with the price or advertising variable to assess the potential endogeneity between sales and prices/advertising.

The base model (Model 1) for regression analysis is: $\ln{\left(per\;capita\;SLT\;sales\right)}_{y,q,m,s}=\beta_{0}+\beta_{1}\ln{\left(own\;prices\right)}_{y,q,m,s}+Y+Q+M+S+\varepsilon_{y,q,m,s}$. The base model estimates the linear relationship between the natural log-transformed per capita sales of each category of SLT products (chewing loose leaf, moist snuff, or snus) measured in ounces (dependent variable) and the natural log-transformed inflation-adjusted average price of the corresponding product (independent variable). By log transforming both sales volume and price, which takes into account the skewed distributions of sales and prices, we can estimate the own-price elasticity from ${\hat{\beta }}_{1}$ directly, assuming constant price elasticity. *Y*, *Q*, *M*, and *S* are the year, quarter, market, and store type fixed effects, respectively.

The natural log of the average price of disposable e-cigarettes (Model 2), the natural log of the average price of cigarettes (Model 3), the natural log of the average price of other SLT products (Model 4), and the natural log of e-cigarette TV ratings (Model 5), were added sequentially to the baseline model to estimate the cross-price elasticities and the association between e-cigarette TV advertising and sales of SLT products.

## 3. Results

### 3.1. Trends of SLT Product Sales Volume and Price

**Figure 1:**
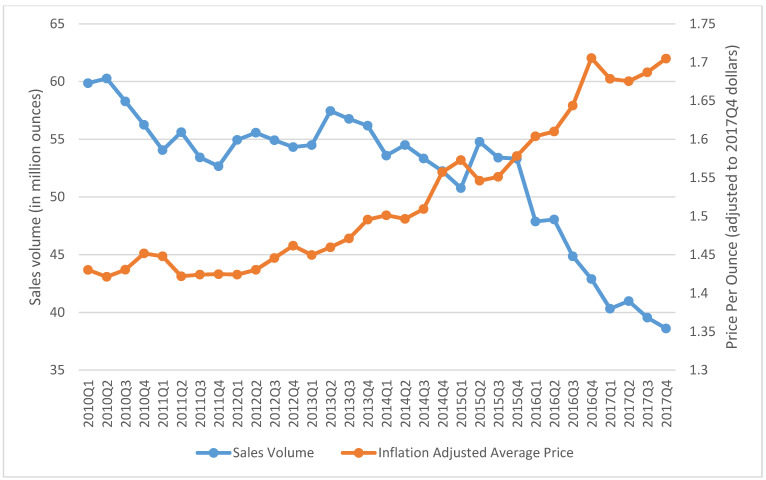
Total retail sales volume in million ounces and average price per ounce adjusted to 2017 Q4 for chewing loose leaf in the US, 2010–2017.

**Figure 2:**
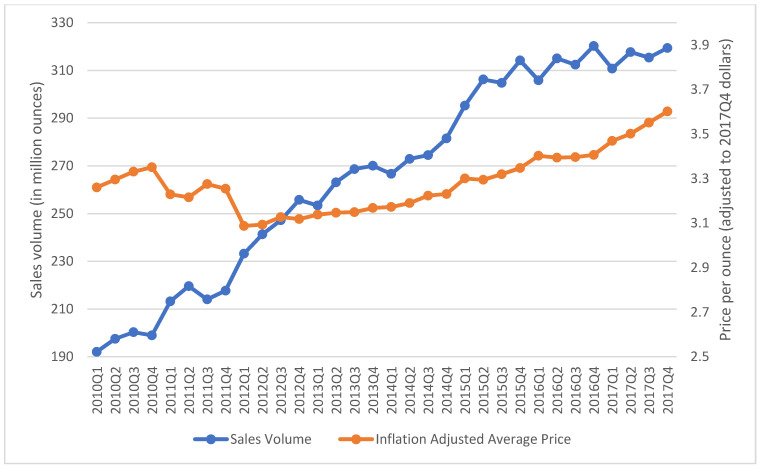
Total retail sales volume in million ounces and average price per ounce adjusted to 2017 Q4 for moist snuff in the US, 2010–2017.

**Figure 3:**
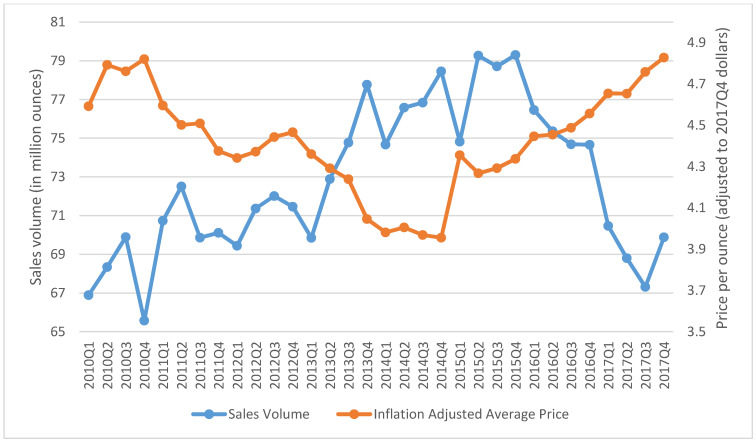
Total retail sales volume in million ounces and average price per ounce adjusted to 2017 Q4 for snus in the US, 2010–2017.

The aggregated quarterly prices and sales of SLT chewing loose leaf, moist snuff, and snus in the US from 2010 Q1 to 2017 Q4 are presented in Figure 1, Figure 2 and Figure 3. During the study period, the inflation-adjusted average price of chewing loose leaf increased from $1.43 per ounce to $1.70 per ounce, and its sales decreased from 59.86 million ounces to 38.61 million ounces (Figure 1). During the same period, the price of moist snuff increased from $3.26 per ounce to $3.60 per ounce, and sales increased from 192.03 million ounces to 319.43 million ounces (Figure 2). The average price of snus decreased from $4.59 per ounce in 2010 Q1 to $3.96 per ounce in 2014 Q4, and then increased to $4.83 per ounce in 2017 Q4. Snus sales increased from 66.90 million ounces in 2010 Q1 to 79.30 million ounces in 2015 Q4, and then decreased to 69.88 million ounces in 2017 Q4 (Figure 3).

Table 1 presents the descriptive statistics for key variables in this study. The average per capita quarterly sales volumes of chewing loose leaf, moist snuff, and snus were 0.05 ounces, 0.29 ounces, and 0.07 ounces, respectively, and the average inflation-adjusted prices for these three categories of SLT products was $2.12 per ounce, $3.49 per ounce, and $4.75 per ounce, respectively. The average price for disposable e-cigarette was $9.24 per piece during the study period. On average, quarterly e-cigarette TV ratings (GRPs) were 531.58 across all markets through the study period. Supplementary Materials, Figure S1 presents the TV ratings at retail market level over time. The e-cigarette TV ratings were low in 2010 and 2011, followed by a rapid increase from 2012 Q3 to 2013 Q2, a period of stagnation with seasonal fluctuations from 2013 Q3 to 2017 Q1, and a sharp decline at the end of 2017. Details about e-cigarette TV advertising exposure in the US can be found in a published paper [32].

### 3.2. Demand for Chewing Loose Leaf

Table 2 presents the regression results from the analysis of chewing loose leaf. The Hausman test results show that the residuals were not significantly associated with the prices of chewing loose leaf (F_1, 2166_ = 0.00, *p*-value = 1.00 for the full model (Model 5)), suggesting that endogeneity was not a major concern in our analysis. In the full model (Model 5) that controlled for the fixed effects of store type, market, year, and quarter; the prices of disposable e-cigarettes, cigarettes, and other categories of SLT products; and e-cigarette TV advertising, the estimated own-price elasticity for chewing loose leaf was −1.83, suggesting that a 1% rise in chewing loose leaf price was associated with an 1.83% reduction in its sales. The estimated cross-price elasticity regarding disposable e-cigarettes was 0.19, indicating that a 1% rise in disposable e-cigarette price was associated with a 0.19% increase in chewing loose leaf sales. However, this cross-price elasticity is not statistically significant. Additionally, results from the full model show that a 1% rise in cigarette price was associated with a 2.13% increase in chewing loose leaf sales. Moreover, a 1% rise in moist snuff price was associated with a 1.72% decrease in chewing loose leaf sales. The cross-price elasticity between snus and chewing loose leaf was estimated to be −0.34; however, it is not statistically significant. The association between e-cigarette TV ratings and chewing loose leaf sales was found to be statistically insignificant.

### 3.3. Demand for Moist Snuff

Table 3 shows the regression results from the analysis of the demand for moist snuff. The results of the Hausman test indicate that moist snuff sales and prices were unlikely to be endogenous (F_1, 2166_ = 0.00, *p*-value = 1.00 for the full model (Model 5)). Estimated coefficients from the full model (Model 5) show that a 1% increase in moist snuff price was associated with a 1.57% decrease in its own sales. A 1% rise in disposable e-cigarette price was found to be associated with a 0.31% increase in moist snuff sales. Additionally, a 1% rise in cigarette price was associated with a 1.33% increase in moist snuff sales, and a 1% rise in snus price was associated with a 1.43% decrease in moist snuff sales. The cross-price elasticity between chewing loose leaf and moist snuff was not statistically significant, neither was the association between e-cigarette TV ratings and moist snuff sales during our study period.

### 3.4. Demand for Snus

Table 4 presents the regression results based on the analysis of the demand for snus. Similar to the analyses of chewing loose leaf and moist snuff, the Hausman test results show that there was insufficient evidence to infer snus sales and prices were endogenous (F_1, 2166_ = 0.00, *p*-value = 1.00 for the full model (Model 5)). The results from the full model (Model 5) show that a 1% rise in snus price was associated with a 2.17% decrease in its own sales. The results of Model 5 also show that a 1% increase in disposable e-cigarette price was associated with a 0.62% increase in snus sales. The associations between cigarette price, chewing loose leaf price, moist snuff price and snus sales were not statistically significant. The association between e-cigarette TV ratings and snus sales was also not significant.

## 4. Discussion

This study is among the first to use market-level aggregated price and sales data to systematically examine whether, and to what extent, e-cigarette sales are associated with the demand for SLT products, controlling for the prices of SLT products, cigarette price, e-cigarette prices and e-cigarette TV advertising ratings, two key influencing factors of e-cigarette sales, and the fixed effects of year, quarter, market, and store type. Specifically, we examined the associations between SLT product prices, cigarette price, disposable e-cigarette price, e-cigarette TV advertising ratings and sales of three categories of SLT products, chewing loose leaf, moist snuff, and snus. Several important findings emerged from our study.

First, we found that one of the strongest factors correlated with SLT product sales was their own prices. The own-price elasticities for all three categories of SLT products were larger than 1 (−1.83 for chewing loose leaf, −1.57 for moist snuff, and −2.17 for snus), indicating that a 1% increase in own price was associated with more than 1% reduction in product sales. This finding suggests that demand for the SLT products was elastic with regard to their own prices, and measures that increase the prices of SLT products could be associated with a substantial decrease in the demand for these products. A previous study, which used the 2007–2014 Nielsen retail scanner data, found that the estimated own-price elasticity was −0.20 for chewing tobacco, −0.63 for moist snuff, and −1.25 for snus [27]. These estimates were smaller in magnitude compared with the estimated own-price elasticities in our study. The large own-price elasticities found in our study may be due to the increased availability of more diversified tobacco and nicotine products in recent years in the US tobacco market, in which tobacco users can choose from a wide variety of products due to the emergence of e-cigarettes, heat-not-burn products, and other tobacco and nicotine products [36,37].

Second, our study revealed the existence of a potential substitution relationship between e-cigarettes and certain SLT products, which has not been previously reported. Specifically, we found that when the price of disposable e-cigarettes increased, the sales of SLT products would increase as well, with the estimated cross-price elasticity being 0.31 for moist snuff and 0.62 for snus, respectively. These results indicate that when disposable e-cigarette prices increase by 1%, sales of moist snuff and snus would increase by 0.31% and 0.62%, respectively. This finding suggests that policies aiming to reduce e-cigarette use by increasing the prices of e-cigarettes may have unintended consequences in terms of increasing the demand for certain SLT products. The cross-price elasticity between e-cigarettes and chewing loose leaf was positive; however, it was not statistically significant. This may reflect the small degree of overlap between users of chewing loose leaf, a more traditional SLT product, who tend to be older in age, and users of e-cigarettes, who tend to be younger. Notably, the association between e-cigarette price and snus sales was the strongest among the three categories of SLT products analyzed in our study. This finding is consistent with results reported in previous studies, which showed that both e-cigarettes and snus were popular among youth and young adults [18,38], who tended to be more price-sensitive than older adult tobacco product users [39,40].

Third, our results revealed that SLT products are potential substitutes for combustible cigarettes, with the estimated cross-price elasticity of 2.13 for chewing loose leaf, and 1.33 for moist snuff. These results indicate that a 1% rise in cigarette price was associated with a 2.13% and 1.33% increase in the demand for chewing loose leaf and moist snuff, respectively. The cross-price elasticity between cigarettes and snus was positive, but not statistically significant. Previous evidence on whether cigarettes and SLT products are substitutes or complements was mixed [41]. For example, a previous study with a design similar to ours did not find a statistically significant relationship between cigarettes and SLT products using the retail sales data from 2007 to 2014, i.e., cigarettes and SLT products were neither substitutes nor complements [27]. Our results suggest that use patterns regarding cigarettes and SLT products may have changed over time, and these products, particularly chewing loose leaf and moist snuff, are increasingly used as substitutes for cigarettes in recent years. Individual-level survey data are needed to further explore the changing perceptions and patterns of use towards cigarettes and SLT products.

Fourth, we also observed a potential complementary relationship across different categories of SLT products. The associations between the price of one category of SLT products and the sales of other categories of SLT products were consistently negative, but they were not always statistically significant. Studies investigating the potential relationships among sub-categories of SLT products are scarce. One study using the Nielsen Household Consumer Panel data from 2004 to 2012 found substitution purchasing behaviors between chewing tobacco and snuff at household level [42]. The different findings may be explained by the differences in the data used: one relying on household purchasing data, and the other relying on retail scanner data. Household data likely under-estimated the sales data due to failures to report by household members. In addition, the differences in findings may also be explained by the changing patterns of use over time due to the availability of new tobacco and nicotine products.

Finally, we found that the association between e-cigarette TV advertising ratings and SLT product sales was not statistically significant. Previous studies reported that e-cigarette TV advertising increased the demand for e-cigarettes [19], reduced the demand for cigarettes [19], and increased the demand for nicotine gums [43]. However, the association between e-cigarette TV advertising and SLT product sales has not been studied before. The insignificant association between e-cigarette TV advertising and SLT product sales may be explained by two reasons. Firstly, although e-cigarette TV advertising is positively associated with e-cigarette sales, the magnitude of its marginal effect on e-cigarette sales is small [19,44]. Since an increase in e-cigarette TV advertising can only elicit small changes in e-cigarette sales, any secondary impact on SLT product sales, if it exists, would be difficult to detect empirically. In addition, other than seasonal fluctuations, which were accounted for by the quarter fixed effects in our analytical models, e-cigarette TV ratings did not vary significantly during the period from 2013 Q2 to 2017 Q1. Lack of significant variations in e-cigarette TV advertising ratings made it difficult to detect its association, if any, with the sales of SLT products. Secondly, although e-cigarettes were gaining popularity and sales increased substantially in recent years, e-cigarette sales still only accounted for less than 5% of the total US tobacco product sales during our study period [4,16]. As such, unlike changes in cigarettes sales, which would generate strong ripple effects across all categories of tobacco products, a rise in e-cigarette sales as a result of an increase in e-cigarette TV advertising, however large it may be, would still represent a small change in total tobacco product sales; hence, it would be unlikely to generate strong ripple effects on other tobacco product sales.

Our study has several limitations. Firstly, our data were limited to the retail price and sales retrieved from the NRS scanner database. Therefore, the estimates only reflect a subset of SLT sales in the US and may not be generalized to the SLT sales through other channels (non-participating retailers, tobacco stores, etc.). Secondly, the prices and sales of SLT products were likely to be endogenous. Several previous studies looking at the impact of e-cigarettes tried to mitigate the potential impact of endogeneity by taking advantage of exogenous changes in either e-cigarette/cigarette state excise taxes or policies regulating these products [42,45]. Unfortunately, during our study period, there were very few changes in either state excise taxes or policies that may have affected the demand for SLT products. However, we did take several measures to mitigate the potential impact of endogeneity between prices and sales. Price and sales measures used in our analyses were constructed at the market level. Since the endogeneity of prices and quantities is presumably strongest at the store- and brand-level, our aggregated price and sales measures can mitigate the potential impact of the endogeneity problem, if it exists [43,46,47]. Importantly, we conducted the Hausman tests to specifically test the existence of endogeneity between prices and sales, with the test results showing that there was insufficient evidence to infer that prices and sales were endogenous in our study. Thirdly, due to data limitations, we were not able to investigate the impact of e-cigarettes on SLT product use at the individual level and explore the potential differential impact across subgroup populations. Future analyses based on individual level data can strengthen this line of research by examining how the growth of e-cigarettes may affect the use of SLT products differentially across population subgroups. Finally, we only analyzed e-cigarette and SLT product retail sales and price data from 2010–2017 due to data availability. The US tobacco and nicotine market has continued to evolve rapidly in recent years. For example, a recent report from the Truth Initiative found that new oral nicotine pouches such as ZYN (a nicotine pouch brand produced by Swedish Match, Stockholm, Sweden), a new flavored nicotine product, were becoming popular among youth [48]. The Truth Initiative’s data showed that 13% of youth and young adults 15–24 years old used nicotine pouches in the past 30 days in fall 2020 [48]. Future studies are needed to examine how these new generations of nicotine products will influence initiation and use of tobacco products both at the aggregated and individual levels.

## 5. Conclusions

Our study found that the demand for SLT products in the US is highly price-sensitive. The positive associations between disposable e-cigarette price and sales of SLT products indicate that certain SLT products are potential substitutes for disposable e-cigarettes. In addition, certain SLT products are also potential substitutes for cigarettes as evidenced by the positive cross-price elasticity between the two. Furthermore, there is some evidence that subcategories of SLT products are potential complements for each other. Our study results suggest that policies that aim to reduce use of cigarettes and e-cigarettes by increasing their prices may need to consider their link with SLT sales due to the potential substitutions between cigarettes/e-cigarettes and certain SLT products.

## Figures and Tables

**Table 1:** Descriptive statistics: per capita sales volumes, inflation-adjusted average prices, and e-cigarette TV ratings.

Variable	Mean	SD ^1^	Min	Max
Per capita chewing loose leaf sales volume	0.05	0.10	0.00	0.84
Per capita chewing loose leaf sales volume (in log)	−4.13	1.84	−13.90	−0.18
Per capita moist snuff sales volume	0.29	0.46	0.00	3.28
Per capita moist snuff sales volume (in log)	−2.72	2.10	−13.02	1.19
Per capita snus sales volume	0.07	0.11	0.00	0.69
Per capita snus sales volume (in log)	−4.25	2.24	−15.59	−0.37
Average price of chewing loose leaf ($ per ounce)	2.12	0.68	0.35	4.42
Average price of chewing loose leaf (in log)	0.70	0.32	−1.04	1.49
Average price of moist snuff ($ per ounce)	3.49	1.03	1.79	7.30
Average price of moist snuff (in log)	1.21	0.29	0.58	1.99
Average price of snus ($ per ounce)	4.75	1.57	2.40	24.07
Average price of snus (in log)	1.52	0.27	0.88	3.18
Average price of disposable e−cigarette ($ per piece)	9.24	1.85	2.96	29.94
Average price of disposable e−cigarette (in log)	2.21	0.18	1.09	3.40
Average price of cigarette ($ per stick)	0.30	0.07	0.18	0.53
Average price of cigarette (in log)	−1.23	0.21	−1.71	−0.64
E−cigarette TV ratings	531.58	512.01	1.00	2553.59
E−cigarette TV ratings (in log)	5.15	2.10	0.00	7.85

^1^ SD: standard deviation.

**Table 2:** Estimated associations between per capita sales of chewing loose leaf and prices of chewing loose leaf, disposable e-cigarettes, cigarettes, moist snuff, snus, and the e-cigarette TV advertising ratings.

	Model 1	Model 2	Model 3	Model 4	Model 5
Chewing loose leaf price	−1.726 ***	−1.885 ***	−2.041 ***	−1.833 ***	−1.833 ***
(in log)	(0.362)	(0.435)	(0.473)	(0.477)	(0.478)
Disposable e-cigarette price		0.211	0.133	0.186	0.186
(in log)		(0.158)	(0.147)	(0.145)	(0.145)
Cigarette price			1.204 **	2.125 ***	2.125 ***
(in log)			(0.555)	(0.568)	(0.568)
Moist snuff price				−1.719 ***	−1.719 ***
(in log)				(0.597)	(0.597)
Snus price				−0.343	−0.343
(in log)				(0.306)	(0.307)
E−cigarette TV rating					0.000
(in log)					(0.003)
Observations	2541	2169	2169	2168	2168
R−squared	0.946	0.949	0.950	0.954	0.954

Note: The outcome is per capita chewing loose leaf sales volume measured by ounce. Fixed effects of store type, market, year, and quarter were included in each model. Standard errors were clustered at the level of store type by market level. *** *p* < 0.01, ** *p* < 0.05.

**Table 3:** Estimated associations between per capita sales of moist snuff and prices of moist snuff, disposable e-cigarettes, cigarettes, chewing loose leaf, snus, and the e-cigarette TV advertising ratings.

	Model 1	Model 2	Model 3	Model 4	Model 5
Moist snuff price	−1.049	−1.370 **	−1.587 **	−1.574 ***	−1.572 ***
(in log)	(0.699)	(0.666)	(0.716)	(0.564)	(0.564)
Disposable e-cigarette price		0.329 **	0.290 *	0.304 **	0.305 **
(in log)		(0.156)	(0.152)	(0.116)	(0.117)
Cigarette price			0.697	1.328 ***	1.328 ***
(in log)			(0.568)	(0.486)	(0.486)
Chewing loose leaf price				−0.243	−0.243
(in log)				(0.306)	(0.306)
Snus price				−1.435 ***	−1.433 ***
(in log)				(0.311)	(0.312)
E-cigarette TV rating					0.003
(in log)					(0.002)
Observations	2571	2195	2195	2168	2168
R-squared	0.963	0.965	0.966	0.968	0.968

Note: The outcome is per capita moist snuff sales volume measured by ounce. Fixed effects of store type, market, year, and quarter were included in each model. Standard errors were clustered at the level of store type by market level. *** *p* < 0.01, ** *p* < 0.05, * *p* < 0.1.

**Table 4:** Estimated associations between per capita sales of snus and prices of snus, disposable e-cigarettes, cigarettes, chewing loose leaf, moist snuff, and the e-cigarette TV advertising ratings.

	Model 1	Model 2	Model 3	Model 4	Model 5
Snus price	−1.971 ***	−2.111 ***	−2.174 ***	−2.177 ***	−2.174 ***
(in log)	(0.356)	(0.364)	(0.339)	(0.369)	(0.370)
Disposable e-cigarette price		0.587 **	0.555 ***	0.615 ***	0.617 ***
(in log)		(0.238)	(0.206)	(0.202)	(0.202)
Cigarette price			0.431	0.751	0.751
(in log)			(0.931)	(0.835)	(0.835)
Chewing loose leaf price				−0.108	−0.109
(in log)				(0.487)	(0.487)
Moist snuff price				−0.496	−0.494
(in log)				(0.891)	(0.893)
E-cigarette TV rating					0.006
(in log)					(0.003)
Observations	2569	2192	2192	2168	2168
R-squared	0.951	0.953	0.953	0.949	0.949

Note: The outcome is per capita snus sales volume measured by ounce. Fixed effects of store type, market, year, and quarter were included in each model. Standard errors were clustered at the level of store type by market level. *** *p* < 0.01, ** *p* < 0.05.

## Data Availability

Restrictions apply to the availability of these data. Data were obtained from the Nielsen Retail Store (NRS) scanner database (The Nielsen Company, LLC, New York, NY, USA) and the Kantar Media Stradegy^TM^ (Kantar Group, London, UK). Contracts with these companies are needed to have access to these data.

## Supplementary Materials

The following are available online at https://www.mdpi.com/article/10.3390/ijerph18136795/s1, Figure S1: E-cigarette TV ratings in gross rating points (GRPs) at retail market level.
