# Supplementary figures and images for: The Association between E-Cigarette Price and TV Advertising and the Sales of Smokeless Tobacco Products in the USA
Source: Int J Environ Res Public Health. 2021 Jun 24;18(13):6795. doi: 10.3390/ijerph18136795 (PMC8297131; doi:10.3390/ijerph18136795)

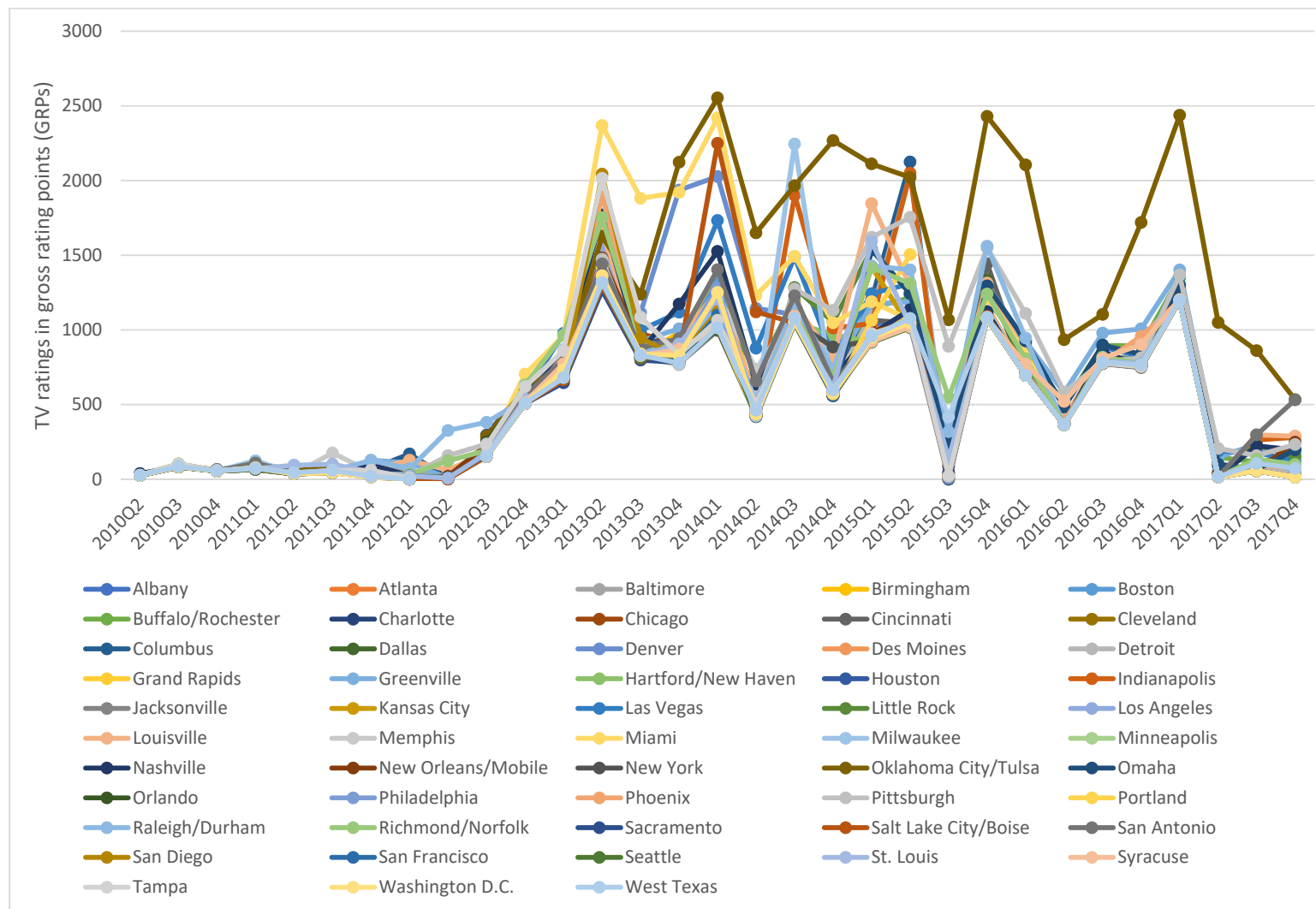

**Figure S1.** E-cigarette TV ratings in gross rating points (GRPs) at retail market level.

Supplement: Supplementary file 1 [file ijerph-18-06795-s001.zip › ijerph-1163501-supplementary.pdf]
